# The Effect of Visual, Spatial and Temporal Manipulations on Embodiment and Action

**DOI:** 10.3389/fnhum.2017.00227

**Published:** 2017-05-04

**Authors:** Natasha Ratcliffe, Roger Newport

**Affiliations:** School of Psychology, University of NottinghamNottingham, UK

**Keywords:** body ownership, sense of agency, MIRAGE, multisensory integration, body representation, visual distortion

## Abstract

The feeling of owning and controlling the body relies on the integration and interpretation of sensory input from multiple sources with respect to existing representations of the bodily self. Illusion paradigms involving multisensory manipulations have demonstrated that while the senses of ownership and agency are strongly related, these two components of bodily experience may be dissociable and differentially affected by alterations to sensory input. Importantly, however, much of the current literature has focused on the application of sensory manipulations to external objects or virtual representations of the self that are visually incongruent with the viewer’s own body and which are not part of the existing body representation. The current experiment used MIRAGE-mediated reality to investigate how manipulating the visual, spatial and temporal properties of the participant’s own hand (as opposed to a fake/virtual limb) affected embodiment and action. Participants viewed two representations of their right hand inside a MIRAGE multisensory illusions box with opposing visual (normal or grossly distorted), temporal (synchronous or asynchronous) and spatial (precise real location or false location) manipulations applied to each hand. Subjective experiences of ownership and agency towards each hand were measured alongside an objective measure of perceived hand location using a pointing task. The subjective sense of agency was always anchored to the synchronous hand, regardless of physical appearance and location. Subjective ownership also moved with the synchronous hand, except when both the location and appearance of the synchronous limb were incongruent with that of the real limb. Objective pointing measures displayed a similar pattern, however movement synchrony was not sufficient to drive a complete shift in perceived hand location, indicating a greater reliance on the spatial location of the real hand. The results suggest that while the congruence of self-generated movement is a sufficient driver for the sense of agency, the sense of ownership is additionally sensitive to cues about the visual appearance and spatial location of one’s own body.

## Introduction

Experiencing a body as one’s own is dependent upon the integration and interpretation of information from various sensory sources. Incoming information from the visual, tactile, vestibular, auditory and proprioceptive systems is integrated to form “bottom-up” contributions to body representation. These must also be interpreted with respect to “top-down” knowledge about the body, which modulates perceptual experience (Tsakiris, 2017). Under normal circumstances, the sense of body ownership seems effortless; that is, we do not have to decide whether or not our body belongs to us. However, experimental paradigms that involve the manipulation of multisensory inputs allow investigation into how this sense of body ownership is formed. In particular, introducing conflict between sensory inputs and top-down knowledge can reveal to what extent each contributes to the sense of owning and controlling the body.

The rubber hand illusion (RHI), first reported by Botvinick and Cohen (1998), has provided much insight into how multisensory interactions contribute to the experience of body ownership. In the basic paradigm, participants watch a rubber hand being stroked at the same time as their unseen real hand is stroked. When the site of stimulation between the two hands is visually congruent and the hands are stroked in synchrony, participants typically report the feeling that the rubber hand starts to become part of their body, and when asked to indicate the location of their real hand, estimates are displaced towards the rubber hand. However when the timing or site of stroking between the real limb and fake hand is asynchronous or incongruent, the illusion is diminished. This finding is replicated throughout the literature (Ehrsson et al., 2004; Tsakiris and Haggard, 2005; Costantini and Haggard, 2007; Shimada et al., 2009) and highlights the importance of intermodal correlations for the experience of body ownership; in this case, the correlation between visual and tactile inputs leads to the experience of ownership over the rubber hand and a modulation of proprioception (Botvinick and Cohen, 1998). Armel and Ramachandran (2003) extend this finding, reporting that synchronous visual and tactile inputs were sufficient to induce a referral of tactile sensations on to a wooden table and furthermore, led to physiological responses consistent with embodiment of the table. The authors suggested that perception is driven by Bayesian inference, implying that so long as stimulation is synchronous, any object may be experienced as belonging to oneself. However, subsequent research has failed to support this assumption, and instead demonstrates that while so-called “bottom-up” sensory correlations are necessary for the illusion, they are not sufficient; “top-down” knowledge constrains the feeling of ownership under certain conditions (Tsakiris and Haggard, 2005). The strength of the illusion is significantly reduced when the rubber hand is replaced with a wooden hand or a block (Tsakiris and Haggard, 2005; Tsakiris et al., 2010a) and when the rubber hand is rotated to an implausible/incongruent posture (Ehrsson et al., 2004; Holle et al., 2011; Ferri et al., 2013). Interestingly, the physical characteristics of the fake hand and their similarity to the participant’s real hand appear to be less crucial; illusion experience is comparable for fake hands of different skin colors (Farmer et al., 2012) and the illusion is maintained for enlarged fake hands (although less so for visually reduced hands; Pavani and Zampini, 2007). Overall, the literature on body-ownership illusions demonstrates that both bottom-up and top-down factors are important in shaping bodily experience. Whilst spatiotemporal correlations between seen and felt stimulation/movements are crucial for the induction of ownership illusions, they are not sufficient; the to-be-integrated stimulus must also be compatible with semantic information about the body (Kilteni et al., 2015). However, the latter component appears somewhat flexible, and under normal conditions, visuo-tactile correlations are able to override some aspects of cognitive knowledge (Farmer et al., 2012; Newport et al., 2015). It is likely that the modification of top-town constraints and the experience of sensory input are bidirectional in nature as the brain attempts to minimize error between predictions and incoming sensory data (see Tsakiris, 2017).

Although the RHI can inform our understanding of some aspects of sensory integration and interaction between bottom-up and top-down components, the traditional paradigm is somewhat restricted, thus limiting what we can infer about how sensory and cognitive factors affect perception of the body. First, the illusion requires the participant to embody a static object, over which they have no motor control. This limits the intermodal correlations that can be investigated; typically only visuo-tactile correspondences are considered as long as the proprioceptive discrepancy is within acceptable limits. Recently, modified versions of the RHI paradigm have emerged that allow a basic motor correspondence between the movements of the participant’s hand and the fake hand (Dummer et al., 2009; Kalckert and Ehrsson, 2012, 2014), but this does not extend to full control over the fake limb. Furthermore, this paradigm required the real and fake hand/fingers to be physically linked, which may affect top-down expectations during the illusion.

Second, the focus of the paradigm is on the application of manipulations to a fake limb, but it is theoretically important to consider how feelings of ownership are affected when these manipulations are applied to the real hand. In the RHI, exploration of the interactions between visual, temporal and spatial properties of the hand is constrained by the possible manipulations that can be applied, and by the requirement that the real hand be hidden from view (vision of the real hand diminishes the illusion; Armel and Ramachandran, 2003). For example, the real hand (based on appearance) can never be moved to an incorrect spatial location, nor can the synchrony between the seen and felt touch on the real hand be manipulated.

Some of these limitations are addressed in the use of virtual reality paradigms, in which participants view a virtual representation of their limb(s) through a head mounted display (Slater et al., 2008). In the virtual hand illusion, the size and position of the virtual hand is programmed such that it appears as though the participant is looking directly at their own hand, and the use of motion tracking technology allows the virtual limb to mimic the participant’s movements. This has enabled more precise investigation into the factors affecting body ownership, with studies investigating the influence of visuomotor correlations, and violations to semantic information including size distortions and body discontinuity (Slater et al., 2009; Sanchez-Vives et al., 2010; Kilteni et al., 2012; Tieri et al., 2015). However, while virtual environments are realistic, the visual characteristics of the limb make it apparent that one is viewing a virtual representation as opposed to one’s own hand. This produces conflict between the existing visual body representation and the seen limb, which may influence interactions between top-down and bottom-up information (Azañón et al., 2016). Since the perceptual aberrations experienced in disorders affecting body representation are misperceptions arising from the real body, it is important to determine whether the principles of body ownership are similar under conditions in which manipulations are applied to the participant’s own hand, i.e., the seen hand matches the existing visual body representation.

Although manipulating the physical properties of the real hand when viewed directly is not possible, manipulations can be applied using video technology. Gentile et al. (2013) manipulated the synchrony and location of seen and felt tactile stimulation using video recordings of the participants’ real hands taken prior to the experiment. Participants viewed the video image through a head-mounted display, creating the impression that they were looking directly at their own hand. However, a drawback of this method is that discrepancies may occur between the pre-recorded video image and the participant’s real hand. The use of pre-recorded videos also limits flexibility in the application of experimental manipulations. These restrictions can be overcome by using a live video image of the participant’s hand. In the video-version of the RHI (the “projected hand illusion (PHI)”; Graham et al., 2015), the rubber hand is replaced by a live video image of the participant’s own hand. As well as allowing precise manipulation of the synchrony of seen and felt brush strokes, the PHI allows the congruency between seen and felt movements to be manipulated. This has been particularly useful for investigating contributions to the sense of agency, including distinctions between active vs. passive movement generation (Tsakiris et al., 2006; Longo and Haggard, 2009; Shimada et al., 2010).

In the PHI, an unmanipulated video image of the participant’s hand is typically either projected onto the surface of a table (e.g., Tsakiris et al., 2006) or shown via a display screen embedded within a table (e.g., Graham et al., 2015). A disadvantage of this set-up is that the viewed hand is in a different plane to the real hand, which may require additional computation factors for the brain to overcome. In addition, by displaying an unmanipulated hand, the top-down factors that can be investigated are restricted. This can be remedied by using more immersive set-ups, such as virtual or mediated reality. The MIRAGE device is an example of such a system, presenting participants with a real-time video image of their own hand that appears in the same spatial location as the participant’s real hand, creating the impression that the participant is viewing their hand directly. This enables visual, spatial and temporal manipulations to be applied concurrently to the participant’s own hand, revealing how such manipulations affect bodily experience.

Previously, Newport et al. (2010) used the MIRAGE to investigate how manipulating the congruency of seen and felt tactile stimulation affected embodiment when participants were presented with two competing representations of the hand. Healthy participants viewed two images of their left hand, and the synchrony of visual information was varied whilst participants engaged in active touch. When one hand was synchronous and the other was not, ownership and reaching movements were consistent with embodiment of the synchronous hand. This finding is consistent with previous literature demonstrating the importance of intermodal correlations in determining ownership. In this study, both hand images were offset at an equal distance away from the participant’s real hand, meaning that spatial (proprioceptive) information was not used to determine ownership. The effect of spatial location was explored in a later study by Newport and Preston (2011), who found that participants disowned the hand in the correct spatial location when feedback was asynchronous, instead taking ownership over the spatially displaced synchronous hand. The manipulation also reduced the accuracy of pointing responses, although reaches were not consistent with complete embodiment of the synchronous hand. Taken together, the findings of these studies demonstrate a strong link between agency and ownership, with ownership of the hand switching with motor synchrony. In both those experiments, however, the appearance of the hand was not manipulated and the two hand images were identical, meaning that only bottom-up contributions to bodily experience were explored. Here, we aim to extend the supernumerary limb paradigm by additionally manipulating the visual appearance of one of the images in order to investigate top-down influences on embodiment. Specifically, we wanted to explore how changing semantic information affects embodiment when manipulations are applied to a realistic representation of the participant’s own limb, rather than a fake hand or virtual limb (Kilteni et al., 2015). By manipulating the congruency of visual, temporal and spatial information of two virtual hands at the same time, the aim is to directly compare the extent to which these factors contribute to body perception and the sense of self. Showing two hands simultaneously, with one always appearing in the same location as the participant’s actual hand, allowed us to explore to what extent certain characteristics “override” others with respect to the sense of embodiment. The question being addressed is whether temporal motor synchrony (and the associated sense of agency) is powerful enough to override top-down visual factors related to ownership of the hand, and whether the addition of congruent proprioceptive information will modulate this. Whereas the previous studies focused on the sense of ownership, the present study aimed to capture a more detailed subjective experience of embodiment by also measuring agency and sense of location (Longo et al., 2008). Along with the inclusion of manual pointing responses, which provided an implicit measure of the “location” component of embodiment, this enabled a more detailed investigation into how sensory manipulations affect different components of embodiment.

Temporal synchrony of movement was predicted to be the strongest driver of embodiment. It was hypothesized that participants would report stronger embodiment over the synchronous hand compared to the asynchronous hand, even when spatial location of the synchronous hand was incongruent, in line with previous findings (Newport et al., 2010; Newport and Preston, 2011). In addition, it was predicted that the experience of embodiment would be modulated by the appearance of the hand, reflecting the influence of top-down knowledge about the body. However, the extent of this modulation was expected to depend on the spatial and temporal properties of the hands, i.e., smaller effect when temporal and spatial information was congruent. Furthermore, visual manipulations were predicted to have a stronger influence on subjective reports compared to pointing responses.

## Materials and Methods

### Participants

Thirty-nine participants (24 female) were recruited using online advertisements and posters. The majority were students at the University of Nottingham. The mean age of participants was 22.12 years (*SD* = 4.05) and 35 self-reported being right handed. This study was carried out in accordance with the recommendations of the School of Psychology ethics committee with written informed consent from all subjects. All subjects gave written informed consent in accordance with the Declaration of Helsinki. The protocol was approved by the School of Psychology ethics committee.

### Apparatus and Procedure

The experiment was carried out using MIRAGE mediated-reality that duplicated a live (delay ~10 ms) digital representation of the participant’s own right hand and presented both of these hands in the same spatial plane as the real hand. Both hands appeared to the right of the body midline and direct view of the upper limb was obscured using a black bib.

Before the experiment began, the participant was given a short time to view his or her unmanipulated and unduplicated right hand in MIRAGE. During this time participants responded to a six-item baseline questionnaire (see Table 1) to verify embodiment of their hand under normal conditions. Responses were on a 7-point scale running from −3 (strongly disagree) to +3 (strongly agree). As expected, all participants immediately reported strong embodiment of the hand in this unmanipulated viewing condition. Following this, participants were given a demonstration of the pointing task (see below), which was demonstrated using two identical images of the participant’s right hand, with no visual distortion applied.

**Table 1:** **Items 1–6 assessed feelings of ownership, agency and sense of location towards each hand (hand on the left vs. hand on the right) resulting in 12 experimental questions**.

	It seemed like…	Category
1	… the hand on the left/right belonged to me	Ownership
2	… the hand on the left/right was part of my body	Ownership
3	… I caused the movement of the left/right hand	Agency
4	… I was in control of the left/right hand	Agency
5	… my hand was in the location where the left/right hand was	Location
6	… when I was tapping, my hand was moving in the location where I saw the left/right hand moving	Location
7	… I had three right hands	Control
8	… I no longer had a right hand	Control

*Two control items (items 7 and 8) were included to check for response bias. Items 1–5 were included in the baseline questionnaire, reworded to refer to the “hand image”. Since there was no tapping in the baseline condition, item 6 was substituted for the following question in the baseline questionnaire: “it seems like the hand in the image is my hand”*.

**Figure 1:**
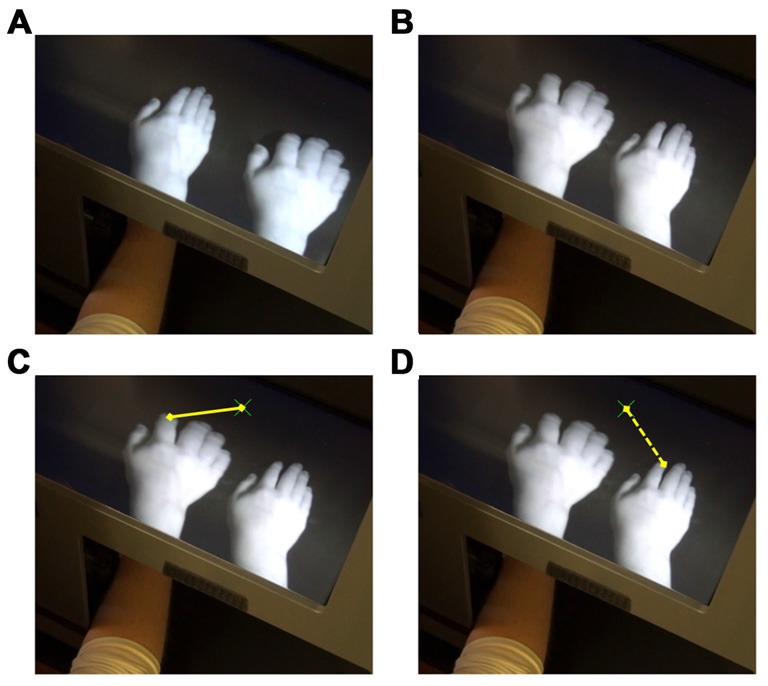
**TOP: example of the appearance and location of the hands in two conditions: (A)** Veridical hand normal; **(B)** Veridical hand distorted. The physical location (real hand on left/right) and synchrony of finger movements (real hand synchronous/asynchronous) was balanced, resulting in a total of eight conditions. In the actual experiment, a black bib occluded vision of the arm. BOTTOM: the yellow lines show example reach trajectories in the pointing task. **(C)** illustrates a reach of the correct distance consistent with perceiving the hand to be in the location of the veridical hand, whilst **(D)** illustrates a reach of the correct distance consistent with perceiving the hand to be in the location of the displaced hand. Note that during the actual task, only the green cross was visible to participants—the hand images were hidden.

At the start of each condition, the MIRAGE-mediated view was blank and the participant placed his or her (unseen) hand inside MIRAGE. The experimenter moved the hand to a specified start location that varied between conditions. A regular short tone, repeated at a rate of ~1 Hz, was played via a computer as a metronome beat and the participant tapped the index finger of the right hand in time to the beat. When tapping in time, the MIRAGE-mediated view presented the participant with two images of his or her right hand. On each trial, the participant saw two hands with opposing characteristics. One hand moved in synchrony with the participant’s movements whilst the other was asynchronous (factor: synchrony). The asynchrony was produced by adding a fixed delay of 500 ms to the video image via software control. At the same time, on the same trial, one hand appeared normal whilst the other was distorted (factor: appearance) and one hand was presented in the same location as the participant’s real hand while the other was presented in a false spatial location, displaced by 12 cm (factor: hand; see Figure 1). After 30 s, the participant stopped tapping the finger and completed either the questionnaire or the pointing task (see below; order randomized across conditions). Following completion, the experimenter picked up the participant’s hand and moved it around before placing it on a start location. To ensure that stimulation was equivalent before each task, the trial was then repeated, with the participant completing the other task after 30 s of tapping.

The distortion was created by defining a region of interest around the hand within the original captured image that was then extracted and transformed to fill a space defined by four new co-ordinates within the workspace. Bicubic filters ensured the smooth transformation of the selected image region in a process that took less than 2 ms with a modal transformation time of 1 ms. The appearance of the distorted hand was selected based on the results of a pilot study in which participants (*N* = 51) rated the appearance of several different hand images. The hand distortion used in the current study was rated as significantly less realistic, less “hand-like” and more distorted than an unmanipulated hand.

To control for the physical location of the real hand (left or right), each condition was presented twice with the physical location of the hand varied such that the false hand appeared either to the left or right of the real hand, but not overlapping, with both hands falling within the right hemispace (see Figure 1). This resulted in a total of eight different conditions, with participants completing the questionnaire and pointing task once for each condition (order randomized). Conditions were completed in a pseudorandom order and counterbalanced between participants. There was a short break between conditions, during which the participant was encouraged to take his or her hand out of MIRAGE and move it around to prevent any stiffness and carry-over effects.

### Questionnaire

At the end of the tapping period, the MIRAGE-mediated view of the hands remained visible. Participants responded to 12 statements designed to assess embodiment of the two hands. The construct of embodiment was based on three distinct components identified by Longo et al. (2008): ownership, agency and location. The items were adapted from those used in previous research (Longo et al., 2008; Tsakiris et al., 2010b) and contained six items relating to embodiment, each asked in reference to the seen left and right hands separately. Two control questions were included to check for response bias, giving a total of 14-items (see Table 1). Participants gave verbal responses to each item using a 7-point scale ranging from −3 (strongly disagree) to + 3 (strongly agree) and the experimenter recorded the response.

### Pointing Task

At the end of the tapping period, the MIRAGE-mediated view of the hands was replaced with a blank workspace except for a green cross located equidistant between the index fingers of the two (now unseen) hands. The participant’s task was to reach, in one smooth movement, and point to the green cross using the index finger such that the finger (if visible) would land at the center of the cross. Reaching movements were recorded via the MIRAGE device.

### Analysis

Participants gave separate questionnaire responses for each hand. A mean score for each component of embodiment (ownership, agency and location), and the control questions, was calculated by averaging each participant’s responses across the relevant items (note that collating scores from individual items in this way produces interval data; see Carifio and Perla, 2008). Positive scores for ownership/agency indicate that the participant experienced a sense of ownership/agency over the specified hand. Positive scores for location indicate that the participant felt as though their hand was in the location of the specified seen hand. Preliminary analysis showed no effect of physical location. Therefore the eight conditions were averaged across physical location (left/right), resulting in four conditions that describe the synchrony (synchronous vs. asynchronous) and appearance (normal vs. distorted) of each hand (the veridical hand, i.e., the hand in the same spatial location as the participant’s actual hand, and the displaced hand, i.e., the hand in a different spatial location to the participant’s actual hand). For brevity, the conditions are referred to by reference to the synchrony and appearance of one hand, (e.g., veridical hand synchronous and normal), but note that the characteristics of the other hand are simply the opposite (in this case, the displaced hand is asynchronous and distorted). The data were analyzed using 3-way repeated measures analysis of variances (ANOVAs) with the factors HAND (Veridical; Displaced), SYNCHRONY (Synchronous; Asynchronous) and APPEARANCE (Normal, Distorted). A potential consequence of analyzing questionnaire data this way is that the assumption of normality of residuals is violated. Exploration of the data from each of the measures showed that the distribution of residuals significantly differed from normal in several conditions (ownership: 4/8; agency: 4/8; location: 3/8; pointing: 1/4). One option was to transform the data, although this would have made the data difficult to interpret. Given the factorial design of the study, non-parametric analysis was considered unsuitable due to the inability of such procedures to investigate interactions between factors. The 3-way design would also have required a large number of *post hoc* comparisons, which after correction for multiple comparisons would dramatically reduce the likelihood of detecting true significant effects (increased type II error). Furthermore, a number of stimulation studies have concluded that ANOVA is “robust” to deviations from normality, particularly when sample size and variance is equal across groups (Glass et al., 1972; Harwell, 1992; Norman, 2010; Schmider et al., 2010). Therefore, the decision was made to proceed with the ANOVA analysis, taking care to interpret statistical findings with respect to measures of central tendency, the spread of the data and effect sizes. In addition to the means displayed in figures, median scores are presented in Table 2 for comparison.

**Table 2:** **Median and interquartile range for ownership, agency and location scores for each hand in each condition**.

Hand	Synchrony	Appearance	Ownership		Agency		Location	
			Median	IQR	Median	IQR	Median	IQR
Veridical	Sync	Normal	2.75	1.00	2.75	0.50	2.50	0.75
		Distorted	1.50	2.00	2.50	1.00	2.00	1.00
	Async	Normal	0.50	2.00	0.50	2.25	0.50	2.25
		Distorted	−1.50	1.75	−0.50	3.00	0.00	2.25
Displaced	Sync	Normal	−2.00	1.50	−0.50	2.25	−2.00	1.25
		Distorted	−0.75	2.25	−0.25	2.50	−1.75	1.25
	Async	Normal	0.50	2.75	2.00	0.75	0.00	2.00
		Distorted	2.00	1.75	2.50	1.00	0.75	2.75

Additionally, to ascertain whether or not participants reported positive experience of each component, one-sample *t*-tests were conducted to test whether means in each condition were significantly greater than zero (Bonferroni method used to control family-wise error rate). This procedure was to ensure that positive ratings for each component represented a meaningful rating of ownership/agency/location.

Reaches made during the pointing task were recorded via video and data was extracted offline using a LabVIEW script that identified the *x*-coordinates of the finger start location, finger endpoint and the target, in pixels. The difference between the finger start point and finger endpoint was calculated with respect to the target location and converted into centimeters (1 cm = 13 pixels), resulting in either a positive or negative value that indicated both the distance and direction of the reach. The distance between the veridical finger and the target was 6 cm. Therefore, a reach of 6 cm indicates a reach of the correct distance consistent with reaching “with” (i.e., from the location of) the veridical hand. Alternatively, a value of −6 cm indicates a reach of the correct distance consistent with reaching “with” (from the location of) the displaced hand (see Figure 1). As with the questionnaire measure, the eight conditions were averaged across left/right location, resulting in four conditions. Again, these are referred to in terms of the characteristics of the real hand in each condition (false hand the opposite). The data were analyzed using a 2-way repeated measures ANOVA with the factors SYNCHRONY (Synchronous; Asynchronous) and APPEARANCE (Normal, Distorted).

## Results

### Ownership

**Figure 2:**
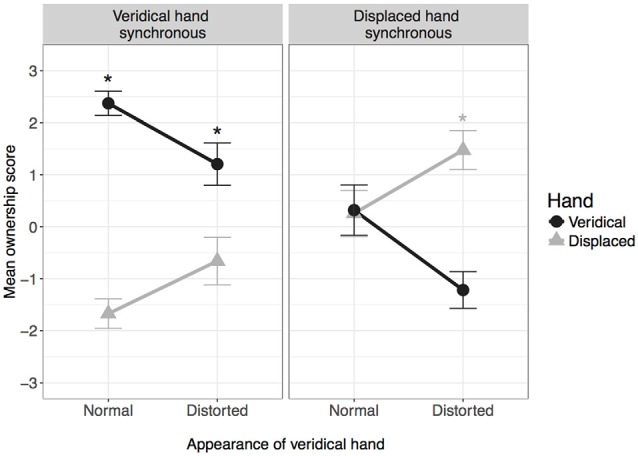
**Mean ownership score in each condition**. *Indicates mean is significantly greater than zero after correcting for multiple comparisons. Error bars: 95% CI.

Figure 2 shows the mean ownership score for each condition. The analysis revealed a significant effect of hand, *F*_(1,38)_ = 23.47, *p* < 0.001, $\eta_{\text{p}}^{2}$ = 0.382, as well as significant two-way interactions for hand by synchrony, *F*_(1,38)_ = 100.70, *p* < 0.001, $\eta_{\text{p}}^{2}$ = 0.72, and hand by appearance, *F*_(1,38)_ = 47.01, *p* < 0.001, $\eta_{\text{p}}^{2}$ = 0.553. Simple main effects analysis comparing hand at each level of synchrony showed that when the veridical hand was synchronous, ownership scores were higher for the veridical hand compared to the displaced hand, *F*_(1,38)_ = 149.70, *p* < 0.001, $\eta_{\text{p}}^{2}$ = 0.798 (*M [SE]* veridical vs. displaced: 1.78 [0.16] vs. −1.17 [0.16]). This pattern was reversed when the veridical hand was asynchronous, *F*_(1,38)_ = 19.29, *p* < 0.001, $\eta_{\text{p}}^{2}$ = 0.337 (*M [SE]* veridical vs. displaced: −0.45 [0.16] vs. 0.87 [0.19]).

Simple main effects analysis comparing hand at each level of appearance showed that when the veridical hand was normal in appearance, ownership scores were higher for the veridical hand compared to the displaced hand, *F*_(1,38)_ = 79.54, *p* < 0.001, $\eta_{\text{p}}^{2}$ = 0.677 (*M [SE]* veridical vs. displaced: 1.35 [0.11] vs. −0.71 [0.18]). However, there was no difference in ownership scores when the veridical hand was distorted, *F*_(1,38)_ = 2.48, *p* = 0.124, $\eta_{\text{p}}^{2}$ = 0.061 (*M [SE]* veridical vs. displaced: −0.01 [0.17] vs. 0.41 [0.16]).

In addition, one-sample *t*-tests were conducted to determine in which conditions ownership scores were significantly greater than zero, indicating a positive experience of ownership. Scores for the veridical hand were significantly bigger than zero in both conditions for which the veridical hand was synchronous (normal: *t*_(38)_ = 20.30, *p* < 0.001; distorted: *t*_(38)_ = 5.22, *p* < 0.001). Scores for the displaced hand were only significantly bigger than zero when the veridical hand was asynchronous and distorted (*t*_(38)_ = 7.54, *p* < 0.001).

In summary, ownership was expressed for the veridical hand when it was synchronous and either of normal or distorted appearance, and for the displaced hand when it was synchronous and normal in appearance.

### Agency

**Figure 3:**
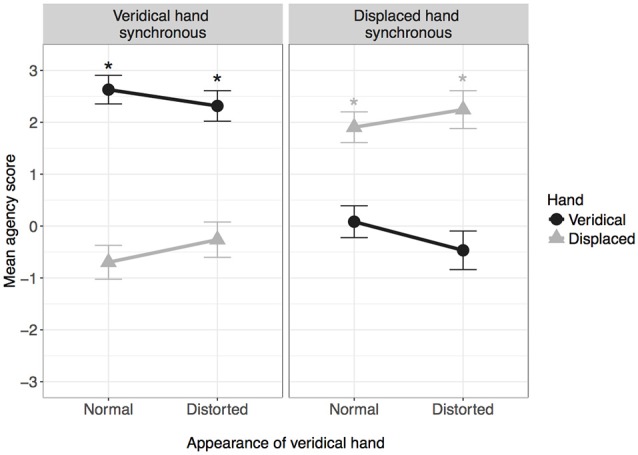
**Mean agency score in each condition**. *Indicates mean is significantly greater than zero after correcting for multiple comparisons. Error bars: 95% CI.

Figure 3 shows agency scores for each condition. The analysis revealed a significant effect of hand, *F*_(1,38)_ = 13.96, *p* = 0.001, $\eta_{\text{p}}^{2}$ = 0.269, along with a significant hand by synchrony interaction, *F*_(1,38)_ = 115.85, *p* < 0.001, $\eta_{\text{p}}^{2}$ = 0.753, and a hand by appearance interaction, *F*_(1,38)_ = 20.22, *p* < 0.001, $\eta_{\text{p}}^{2}$ = 0.347.

Simple main effects analysis comparing hand at each level of synchrony showed that when the veridical hand was synchronous, agency scores were higher for the veridical hand, *F*_(1,38)_ = 126.39, *p* < 0.001, $\eta_{\text{p}}^{2}$ = 0.769 (*M [SE]* veridical vs. displaced: 2.47 [0.09] vs. −0.48 [0.24]). The reverse pattern was observed when the veridical hand was asynchronous, *F*_(1,38)_ = 78.50, *p* < 0.001, $\eta_{\text{p}}^{2}$ = 0.674 (*M [SE]* veridical vs. displaced: −0.19 [0.21] vs. 2.07 [0.12]).

Simple main effects analysis comparing hand at each level of appearance showed that when the veridical hand was normal, agency scores were higher for the veridical hand compared to the displaced hand, *F*_(1,38)_ = 35.66, *p* < 0.001, $\eta_{\text{p}}^{2}$ = 0.48 (*M [SE]* veridical vs. displaced: 1.36 [0.11] vs. −0.60 [0.15]). However, there was no difference in agency scores when the veridical hand was distorted, *F*_(1,38)_ = 0.26, *p* = 0.615, $\eta_{\text{p}}^{2}$ = 0.007 (*M [SE]* veridical vs. displaced: 0.92 [0.13] vs. 0.99 [0.14]).

One-sampled *t*-tests revealed that agency scores for the veridical hand were significantly bigger than zero in both conditions for which the veridical hand was synchronous (normal: *t*_(38)_ = 32.22, *p* < 0.001; distorted: *t*_(38)_ = 20.87, *p* < 0.001). Furthermore, agency scores for the displaced hand were significantly bigger than zero in both conditions for which the veridical hand was asynchronous i.e., the displaced hand was synchronous (normal: *t*_(38)_ = 14.71, *p* < 0.001; distorted: *t*_(38)_ = 14.99, *p* < 0.001).

In summary, agency was expressed for the hand that was in temporal synchrony with the movements of the veridical hand, regardless of location or appearance.

### Location

**Figure 4:**
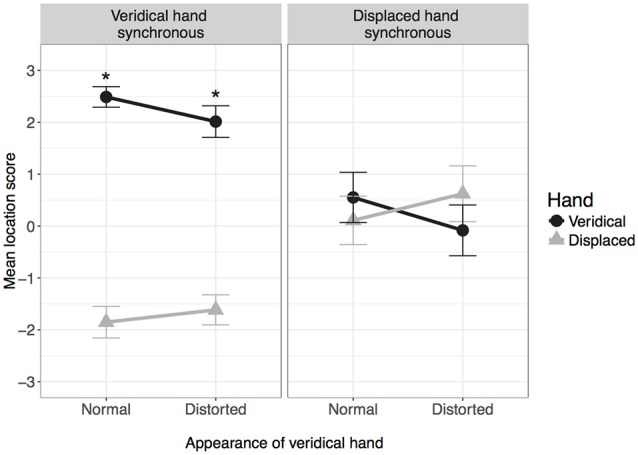
**Mean location score in each condition**. *Indicates mean is significantly greater than zero after correcting for multiple comparisons. Error bars: 95% CI.

Figure 4 shows location scores for each condition. Again, there was a main effect of hand, *F*_(1,38)_ = 60.93, *p* < 0.001, $\eta_{\text{p}}^{2}$ = 0.62, and significant two-way interactions for hand by synchrony, *F*_(1,38)_ = 88.71, *p* < 0.001, $\eta_{\text{p}}^{2}$ = 0.700, and hand by appearance, *F*_(1,38)_ = 19.65, *p* < 0.001, $\eta_{\text{p}}^{2}$ = 0.341.

Simple main effects analysis comparing hand at each level of synchrony showed that when the veridical hand was synchronous, location scores were significantly higher for the veridical hand, *F*_(1,38)_ = 363.78, *p* < 0.001, $\eta_{\text{p}}^{2}$ = 0.905 (*M [SE]* veridical vs. displaced: 2.25 [0.09] vs. −1.73 [0.15]). However, there was no difference in location scores when the veridical hand was asynchronous, *F*_(1,38)_ = 0.100, *p* = 0.754, $\eta_{\text{p}}^{2}$ = 0.003 (*M [SE]* veridical vs. displaced: 0.23 [0.20] vs. 0.37 [0.23]).

Simple main effects analysis comparing hand at each level of appearance showed that when the veridical hand was normal, location scores were higher for the veridical hand compared to the displaced hand, *F*_(1,38)_ = 90.67, *p* < 0.001, $\eta_{\text{p}}^{2}$ = 0.705 (*M [SE]* veridical vs. displaced: 1.52 [0.12] vs. −0.87 [0.16]). The reverse pattern was observed when the veridical hand was distorted, *F*_(1,38)_ = 26.46, *p* < 0.001, $\eta_{\text{p}}^{2}$ = 0.411 (*M [SE]* veridical vs. displaced: 0.97 [0.14] vs. −0.49 [0.17]).

One-sampled *t*-tests revealed that location scores for the veridical hand were significantly bigger than zero in both conditions for which the veridical hand was synchronous (normal: *t*_(38)_ = 29.92, *p* < 0.001; distorted: *t*_(38)_ = 15.27, *p* < 0.001). When the veridical hand was asynchronous, location scores were not significantly greater than zero for either the normal or distorted hand after correcting for multiple comparisons.

In summary, participants felt as though their hand was in the same location as the veridical hand regardless of whether it appeared normal or distorted, but only when the veridical hand was synchronous; when it was asynchronous (and thus the displaced hand was synchronous), the perceived location of the hand was ambiguous.

### Pointing Task

**Figure 5:**
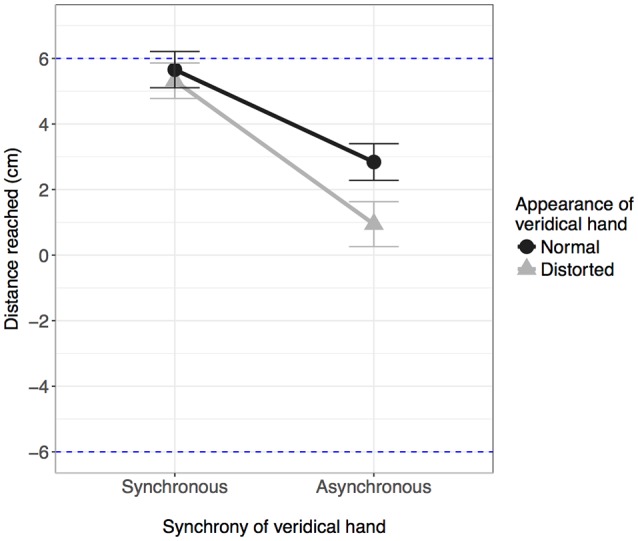
**Mean distance reached (cm) in the pointing task (in horizontal plane)**. The dotted line at 6 cm represents a reach of the correct distance consistent with reaching “with” (i.e., from the location of) the veridical hand. Alternatively, the dotted line at −6 cm indicates a reach of the correct distance consistent with reaching “with” (from the location of) the displaced hand. A reach of 0 cm indicates that the participant reach straight-ahead, consistent with the perception that their hand was located directly between the two hand images they saw. Error bars: 95% CI.

Mean distance reached (cm) is displayed in Figure 5, with lower values reflecting reduced accuracy. The analysis revealed a main effect of synchrony, *F*_(1,37)_ = 75.72, *p* < 0.001, $\eta_{\text{p}}^{2}$ = 0.672, and a main effect of appearance, *F*_(1,37)_ = 49.95, *p* < 0.001, $\eta_{\text{p}}^{2}$ = 0.574, as well as a significant interaction between the two, *F*_(1,37)_ = 11.55, *p* = 0.002, $\eta_{\text{p}}^{2}$ = 0.238.

Simple main effects analysis of appearance at each level of synchrony revealed a significant effect of appearance both when the veridical hand was synchronous, *F*_(1,37)_ = 6.44, *p* = 0.016, $\eta_{\text{p}}^{2}$ = 0.148, and when it was asynchronous, *F*_(1,37)_ = 31.78, *p* < 0.001, $\eta_{\text{p}}^{2}$ = 0.462. When the veridical hand was synchronous, accuracy was lower when the hand was distorted compared to when it was normal (mean difference: 0.47 cm), although it should be noted that overall accuracy remained high. The same pattern was observed when the veridical hand was asynchronous, with lower accuracy for when the veridical hand was distorted compared to when it appeared normal, although the difference between the means was much larger compared to when the veridical hand was synchronous (mean difference: 1.95 cm), and overall accuracy was lower.

In summary, when the synchronous hand was proprioceptively congruent with the real hand, participants pointed with the synchronous hand (that is, from the location of the synchronous hand) regardless of physical appearance. When the synchronous hand was incongruent with the location of the real hand, the inferred origin of reaches was shifted towards the displaced, synchronous hand, but not completely. This effect was modulated by appearance and was greater when the displaced, synchronous hand was normal in appearance.

### Correlations between Components

In addition to the main analyses, exploratory correlational analyses were conducted to assess the relationship between the different components of embodiment in each condition. For the subjective components, correlations were conducted on scores for the veridical hand only.

Pearson correlations (shown in Table 3) revealed significant positive correlations between the subjective components, ownership, agency and location, in all conditions except when the veridical hand was synchronous and distorted (displaced hand asynchronous and normal). In that condition, location scores were significantly correlated with both ownership and agency, however ownership and agency were not significantly correlated with each other.

**Table 3:** **Pearson correlations between the four measures for each condition**.

	Ownership	Agency	Location

**Veridical hand synchronous and normal**
Ownership	−		
Agency	0.404*	−	
Location	0.421**	0.504**	−
Pointing	−0.060	0.251	0.331*
**Veridical hand synchronous and distorted**
Ownership	−		
Agency	0.178	−	
Location	0.382*	0.792**	−
Pointing	0.100	0.200	0.242
**Veridical hand asynchronous and normal**
Ownership	−		
Agency	0.452**	−	
Location	0.727**	0.490**	−
Pointing	0.458**	0.045	0.577**
**Veridical hand asynchronous and distorted**
Ownership	−		
Agency	0.494**	−	
Location	0.489**	0.545**	−
Pointing	−0.079	−0.116	0.326**

Significant associations are indicated by asterisks, where *p < 0.05, **p < 0.01. N = 39 for all.

Pointing accuracy was significantly correlated (positively) with location scores for all conditions except when the veridical hand was synchronous and distorted. Aside from a significant correlation between pointing accuracy and ownership scores in one condition, all other correlations were not significant (see Table 3).

## Discussion

This study investigated how manipulating visual, temporal and spatial information about the hand influenced body perception in relation to agency and ownership. Participants tapped the index finger up and down whilst viewing two representations of his or her own hand that had opposing visual (normal or distorted), temporal (synchronous or asynchronous movement) and spatial (real location or false location) characteristics. Questionnaire responses captured perceived ownership, agency and sense of location for each hand, and pointing responses served as an implicit measure of embodiment.

For agency, the strongest driver was temporal synchrony; participants felt a sense of control over whichever hand moved in synchrony with their own movements, and the sense of agency remained regardless of visual appearance or location relative to the real hand (see Figure 3). The effects were somewhat different with regards to ownership; whilst temporal synchrony remained an important factor, the sense of ownership was additionally modulated by visual appearance and location: while ownership was reported for the synchronous hand in either location, the strength of feeling was lessened if that hand was grossly distorted or if the location was incongruent with the real hand (see Figure 2), to the extent that ownership was not claimed if the hand was both distorted AND in an incongruent location. Subjectively, the real hand was felt to be in the same location as the synchronous hand only when the synchronous hand was in the same location as the real hand in reality (veridical hand synchronous conditions in Figure 4). When the asynchronous hand was in the same location as the real hand in reality (veridical hand asynchronous conditions in Figure 4), subjective location became uncertain and was reported to be in neither the location of the synchronous nor asynchronous hand. Subjective reports were consistent with objective pointing data: the inferred start locations were consistent with reaching “with” (from the location of) the synchronous hand when it was in the same physical location as the real hand, but from between the two hands when the hand seen in the same location as the real hand was asynchronous (see Figure 5). That is, start locations were dragged towards the synchronous hand, but not completely, suggesting modulation by proprioception and congruence with the real hand location.

The present work is the first study to investigate how distorting the appearance of the participant’s own hand affects embodiment when simultaneous spatial and/or temporal manipulations are applied. Previous experiments have demonstrated how manipulating the appearance, temporal synchrony or the spatial location of fake or virtual limbs influences feelings of embodiment. However, it is unclear whether the same mechanisms apply when manipulations are applied to (an image of) one’s own hand. Experiencing an external object as part of your own body is likely to involve different multisensory interactions compared to when manipulations are applied to an image of one’s own hand. In comparison to a fake hand, viewing a representation of one’s own hand might be expected to evoke stronger top-down influences, due to the fact that the visual appearance of the hand is consistent with the visual representation in the existing internal model (Tsakiris, 2010). This may influence the integration of top-down and bottom-up inputs, potentially causing visual information regarding the appearance of the hand to be given a stronger weighting compared to situations in which visual information is obviously inaccurate or false. Such strong visual information may even be sufficient to overcome other sensory discrepancies, such as incongruence between seen and felt movements (or touch), contradicting results from fake/virtual body paradigms. Such a finding would also have important implications for our understanding of disorders of body representation, where perceptual aberrations arise from one’s own body rather than misperceptions of external objects.

Importantly, the findings show that even when participants view normal and distorted representations of their own hand, visuomotor synchrony is the strongest driver of both agency and ownership: in all four conditions, participants reported a strong sense of agency for whichever hand was synchronous, and a sense of ownership was reported over the synchronous hand in all but one condition. However, the results also show that violations of top-down knowledge about the body, introduced through the visual distortion, have different implications for agency and ownership. While the contrasting visual appearance between the two hands had little effect on agency, participants reported significantly less ownership over the hand when it was distorted, indicating that ownership is more influenced by the visual information about the hand form/appearance compared to agency. This is in line with findings showing that postural manipulations of fake/virtual hands have a greater effect on ownership compared to agency (Kalckert and Ehrsson, 2012; Salomon et al., 2016). Here, we extend these findings by showing a similar effect when manipulating the visual characteristics of the participant’s own hand, indicating that agency and ownership are at least partially independent processes. Further evidence for this is found in the comparison of ownership and agency ratings in the condition for which the veridical hand was both asynchronous and of normal appearance (displaced hand synchronous-distorted); participants reported a strong sense of agency over the displaced hand but no sense of ownership over either hand. This demonstrates first that ownership is not necessary for agency, and second that agency is not sufficient for ownership, supporting previous suggestions that ownership and agency are dissociable (Tsakiris et al., 2010b; Kalckert and Ehrsson, 2012). However, the present results cannot conclude that agency and ownership are completely independent. It is notable that participants did not report ownership for a hand that they did not also have a sense of agency for although see Kalckert and Ehrsson (2012), and furthermore under no circumstances did participants claim ownership of one hand, but agency of another.

The significant associations between the subjective experiences of ownership, agency and location support the notion that these three factors can be considered as subcomponents of a broader bodily experience, termed embodiment (Longo et al., 2008; Graham et al., 2015). In particular, the strong association between ownership and agency suggests that these two components share at least some common mechanisms. Although both ownership and agency correlated with the subjective sense of location, neither correlated with pointing accuracy, the implicit measure of perceived hand location (with the exception of ownership in one condition). However, pointing accuracy did correlate with subjective location scores in all but one condition, supporting the suggestion that the pointing task provides an implicit measure of the location component of embodiment.

The current study extends previous findings from the supernumerary limb paradigm, which found that ownership of the hand moved with visuomotor synchrony (Newport et al., 2010; Newport and Preston, 2011). However, in those experiments, the two hands were identical in appearance. The current experiment demonstrates that visuomotor synchrony does not completely dictate ownership when competing top-down (appearance) and bottom-up (proprioception) factors provide additional, conflicting information. Similarly, ownership is not driven by appearance alone; seeing a representation of one’s own hand (normal appearance) is not sufficient to override the influences of incongruent temporal and spatial information. Rather unsurprisingly, perhaps, the brain seems to weigh up the available sensory information and make sense of the body accordingly. When the hand is synchronous, in the same location and with veridical appearance, it is owned; when it is asynchronous, in the wrong location and looks wrong, it is not; all other combinations are somewhere in between.

While the present study demonstrates that altering the appearance of the hand reduces the sense of ownership, participants still experienced ownership over the distorted hand when it was temporally and spatially congruent with their actual limb (see Figure 2, veridical hand synchronous and distorted condition). The extent to which the appearance of the hand can be altered whilst maintaining a sense of ownership (when all other factors are constant) remains unclear. In future work we aim to clarify this by manipulating the visual similarity between the participant’s own hand and the viewed limb in different stages, gradually increasing the level of distortion/dissimilarity. This will shed further light on the interplay between bottom-up and top-down factors during body representation.

Overall, the findings reveal important information about the way in which different sensory information is used to form a representation of the body. Few studies have examined how specific visual characteristics of the hand affect experience of embodiment, despite vision providing a key source of information used to distinguish between self and other. When visual characteristics have been explored, these have been limited to altering the appearance of a fake limb, rather than changing the appearance of the participant’s own hand (e.g., Heed et al., 2011; Bertamini and O’Sullivan, 2014). In line with previous research, the results showed that temporal synchrony had the strongest effect on the sense of ownership, agency and perceived location of the hand as measured by both the questionnaire and pointing responses. Importantly, the visual appearance of the hand also had a smaller but significant effect on responses. The difference between normal and distorted hands was minimal for both agency and location scores, but the effect was larger for ownership scores, suggesting visual information is weighted more strongly in determining the sense of ownership. Visual information also had an effect on pointing responses, but only in conditions for which the veridical hand was asynchronous (displaced hand synchronous). The findings also demonstrate that participants were sensitive to the spatial location of the hands, although the extent to which this affected responses differed across components. Both questionnaire scores of perceived location and pointing responses were particularly sensitive to hand location; specifically, synchronous movement of the displaced hand was not sufficient to result in a shift in perceived hand location towards that hand, even when it also appeared normal.

Taken together, the findings support the distinction between agency and ownership. While visuomotor synchrony is sufficient for the sense of agency, the sense of ownership is reduced when the visual appearance or physical location of the hand is manipulated. Furthermore, the study highlights how distinct sensory inputs are weighted differently, and combined with top-down knowledge of the body, to contribute to individual components of embodiment.

## Author Contributions

NR and RN designed the study, interpreted the data and wrote the manuscript. NR conducted the experiment and analyzed the data.

## Funding

This work was supported by the BIAL Foundation under grant number 203/12.

## Conflict of Interest Statement

The authors declare that the research was conducted in the absence of any commercial or financial relationships that could be construed as a potential conflict of interest.
